# Legg-Calve-Perthes disease

**DOI:** 10.11604/pamj.2021.39.187.30522

**Published:** 2021-07-08

**Authors:** Chanan Goyal, Anshuman Shukla

**Affiliations:** 1 Datta Meghe Institute of Medical Sciences, Wardha, India,; 2 Government Physiotherapy College, Raipur, India

**Keywords:** Legg-Calve-Perthes disease, Perthes disease, femoral head avascular necrosis

## Image in medicine

We report a case of a 9-year-old male who presented with limping gait. The primary concern of the parents was that, he started walking in an awkward fashion two months ago. The birth and developmental history were uneventful. Child complaint of mild inconsistent pain in left hip joint. On observation in standing posture, asymmetry was noted as he was bearing less weight on left lower limb due to pain. On examination, in supine lying, active as well as passive abduction of left hip were terminally limited by pain. When he was made to stand on right leg, pelvis was neutral. On the other hand, trunk shifted to left side while he attempted single leg standing on left leg and it was difficult to maintain balance. Thus, Trendelenburg sign was positive. Trendelenburg gait was evident as the trunk shifted to the stance side. X-ray reveals smaller and compressed femoral epiphyseal size with blurred epiphyseal plate on the left side. Stage I changes in imaging were noted according to Waldenstrom´s classification along with Stulberg class III of Perthes disease. Legg-Calve-Perthes disease is insidious onset idiopathic avascular necrosis of femoral epiphysis in children, commonly between five to eight years of age. Physiotherapy intervention comprised active range of motion of left hip joint in non-weight bearing position, isometric exercise of gluteal muscles, prescription of weight relieving trilateral orthosis, parent and child education about de-loading of hip joint and avoiding high impact activities. Containment surgery for left hip was planned by orthopaedic surgeon.

**Figure 1:**
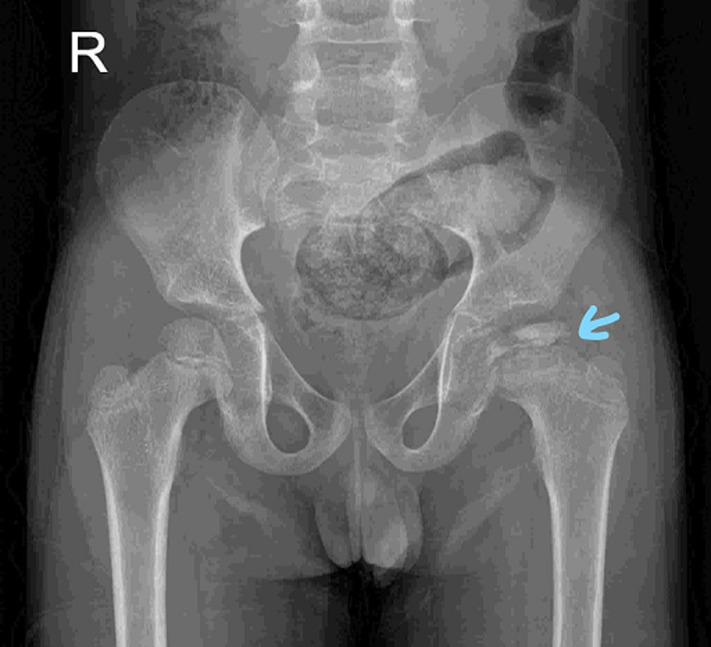
blue arrow shows avascular necrosis of left femoral epiphysis

